# Celecoxib and Etoricoxib may reduce risk of ischemic stroke in patients with rheumatoid arthritis: A nationwide retrospective cohort study

**DOI:** 10.3389/fneur.2022.1018521

**Published:** 2022-10-20

**Authors:** Acer I-Hung Chen, Yung-Heng Lee, Wuu-Tsun Perng, Jeng-Yuan Chiou, Yu-Hsun Wang, Lichi Lin, James Cheng-Chung Wei, Hsi-Kai Tsou

**Affiliations:** ^1^Medical Intensive Care Unit, Ronald Reagan UCLA Medical Center, Los Angeles, CA, United States; ^2^School of Medicine, Chang Gung University, Taoyuan, Taiwan; ^3^Department of Health Services Administration, China Medical University, Taichung, Taiwan; ^4^Department of Public Health, China Medical University, Taichung, Taiwan; ^5^Department of Orthopedics, Cishan Hospital, Ministry of Health and Welfare, Kaohsiung, Taiwan; ^6^Department of Center for General Education, National United University, Miaoli, Taiwan; ^7^Department of Recreational Sport and Health Promotion, National Pingtung University of Science and Technology, Pingtung, Taiwan; ^8^School of Health Policy and Management, Chung Shan Medical University, Taichung, Taiwan; ^9^Department of Medical Research, Chung Shan Medical University Hospital, Taichung, Taiwan; ^10^Department of Statistics, Oklahoma State University, Stillwater, OK, United States; ^11^Division of Allergy, Immunology and Rheumatology, Chung Shan Medical University Hospital, Taichung, Taiwan; ^12^Institute of Medicine, Chung Shan Medical University, Taichung, Taiwan; ^13^Graduate Institute of Integrated Medicine, China Medical University, Taichung, Taiwan; ^14^Functional Neurosurgery Division, Neurological Institute, Taichung Veterans General Hospital, Taichung, Taiwan; ^15^Department of Rehabilitation, Jen-Teh Junior College of Medicine, Nursing and Management, Miaoli, Taiwan; ^16^College of Health, National Taichung University of Science and Technology, Taichung, Taiwan; ^17^Department of Post-baccalaureate Medicine, College of Medicine, National Chung Hsing University, Taichung, Taiwan

**Keywords:** non-steroidal anti-inflammatory drugs (NSAIDs), rheumatoid arthritis, risk, ischemic stroke (IS), Celecoxib-compound CID: 2662, Etoricoxib (CID: 123619)

## Abstract

**Background and purpose:**

Previous studies reported conflicting results about the risk of ischemic stroke associated with the use of non-steroidal anti-inflammatory drugs (NSAIDs) in patients with rheumatoid arthritis (RA). We aimed to investigate two specific COX-2 inhibitors, Celecoxib and Etoricoxib, and their corresponding effects on the risk of ischemic stroke in patients with RA.

**Patients and methods:**

10,857 patients newly diagnosed with RA were identified and sampled from the Taiwanese National Health Insurance Research Database during the period from 2001 to 2009. The identification of RA was based on the criteria of ICD-9-CM diagnosis code 714.0. Patients diagnosed with cerebrovascular disease and those receiving RA treatment prior to the first diagnosis of RA were excluded. Study endpoint was ischemic stroke, defined by ICD-9-CM code. Cox proportional hazard models and Kaplan Meier curves were used to reveal covariates and differences by drugs in the risk of ischemic stroke. Dosages for Celecoxib were defined as ≤ 200 and >200 mg/day; those for Etoricoxib were 0 and >0 mg/day.

**Results:**

Among 7,904 RA patients, 6,669 did not take Celecoxib and 564 (8.46%) of them experienced an ischemic stroke event. Of the 597 individuals who took ≤ 200 mg/day of Celecoxib, 58 (9.72%) had strokes. Of the 638 patients who took >200 mg/day of Celecoxib, 38 (5.96%) eventually experienced a stroke. Among the 7,681 patients who did not take Etoricoxib, 654 (8.51%) experienced an ischemic stroke, while 6 (2.69%) in 223 patients who consumed Etoricoxib had a stroke event. Consuming more than 200 mg of Celecoxib per day for <3.5 years lowered the incidence rate for strokes [hazard ratio (HR) 0.67, 95% Confidence Interval (CI) 0.48–0.93 for dosage and HR 0.22, 95% CI 0.10–0.46 for duration, both *p* < 0.001], while consuming any dosage of Etoricoxib significantly decreases the possibility (HR 0.35, 95% CI 0.16–0.80, *p* < 0.001). On the other hand, consuming Etoricoxib for 8 years might have a neutral or even a potentially protective effect compared to at 3.8 years.

**Conclusion:**

This population-based retrospective cohort study has shown that Celecoxib and Etoricoxib reduce the risk of ischemic stroke in patients with RA in a dose- and time-dependent manner.

## Introduction

The Centers for Disease Control and Prevention reported an incidence of 795,000 new cases per year for both ischemic and hemorrhagic strokes in the United States. Among these, 610,000 are new-onset while the other 185,000 are recurring, meaning that nearly 25% of strokes are in individuals who had experienced a previous stroke. Strokes are also responsible for more than 130,000 deaths per year, representing 5% of all deaths in the United States, making it the fifth leading cause of death (1). Similarly, global lifetime stroke incidence is 24.9% while this number increases to 38.8% for East Asia (2). The genetic and environmental risk factors vary across racial and ethnic groups, with African Americans twice as likely to experience a stroke as well as to die from it, in comparison to Caucasians. The risks for Hispanic populations fall between those of African Americans and Caucasians (3). Although risk is positively correlated with age, strokes can occur at any age (1). Since 87% of all strokes are ischemic strokes, research on the correlations of high blood pressure, high cholesterol, and non-steroidal anti-inflammatory drug (NSAID) use with the incidence rate of stroke could potentially yield important results for future practices in health care.

A review of the literature in general populations reveals two opposing conclusions: either the use of NSAIDs for rheumatoid arthritis (RA) puts one at a greater risk for ischemic stroke, or NSAIDs are neutral or even protective in terms of the risk of ischemic stroke. For instance, an overall review of 75 observational studies concluded that the risk of stroke was increased with the use of two specific NSAIDs: Rofecoxib (4) and Diclofenac (5–10). Another retrospective cohort study in Australia showed that any usage of NSAIDs increased a subject's risk of ischemic stroke 1.88 times, with a 95% Confidence Interval (CI) of 1.70–2.08. In addition, the use of most NSAIDs, especially Indomethacin (11), Rofecoxib (4), and Celecoxib (8, 12), has been found to associate with an increased risk for stroke (13, 14).

Other studies have suggested the opposite results. A meta-analysis to determine the correlation of cyclooxygenase-2 (COX-2) selectivity with cardiovascular risk found that, except for Rofecoxib, other NSAIDs and COX-2 inhibitors had no statistically significant correlation with cardiovascular death, while Celecoxib decreased the incidence rate [hazard ratio (HR) 0.805, 95% CI 0.658–0] (15–20). Furthermore, one of the largest patient-level meta-analyses performed by White et al. concluded from 39 randomized clinical trials that their team was not able to demonstrate a difference between Celecoxib and non-selective NSAIDs in regard to the incidence of cardiovascular events (21).

Celecoxib and Etoricoxib are two selective COX-2 inhibitors approved for complex diseases and disorders such as rheumatoid arthritis, neurodegenerative diseases and cardiovascular disorders (22). Highly selective COX-2 inhibitors have also been researched and shown to halve gastrointestinal toxicity side effects (23). Celecoxib and Etoricoxib therefore have become commonly used in RA patients in Division of Allergy, Immunology and Rheumatology. The main purposes of this cohort study were to determine the risk of first-occurrence ischemic stroke associated with NSAID use and the risk of ischemic stroke associated with the use of two specific COX-2 inhibitor NSAIDs, Celecoxib, and Etoricoxib, in subjects with RA.

## Patients and methods

### Data source

All patient data were extracted from the Taiwan National Health Insurance Research Database (NHIRD), an insurance claims-based dataset composed of de-identified healthcare data of more than 99% of the residential population in Taiwan. The Taiwan NHIRD currently has more than 25 million enrollees. Healthcare information within this database includes outpatient visits, hospitalizations, disease diagnoses, prescriptions and the related medical expenses. The disease diagnosis criteria were based on the International Classification of Diseases, Ninth Revision, Clinical Modification (ICD-9-CM). The diagnosis coding in this dataset is periodically reviewed to assure accuracy. This population-based dataset, with a follow up from 2000 to 2010, is widely used in epidemiological studies, including those for RA and stroke (24–26).

### Patient identification and matching comparison cohort

**Figure 1:**
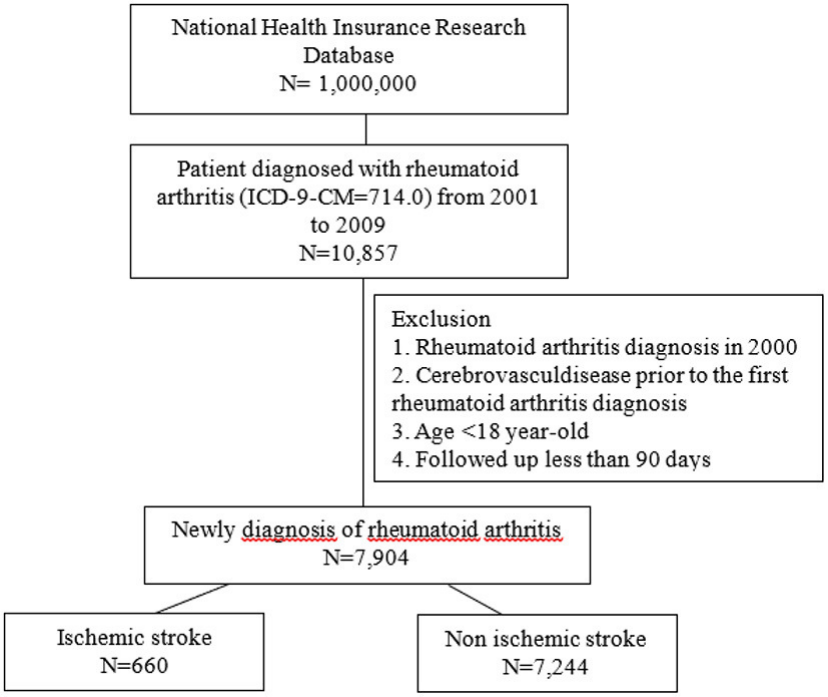
Patient identification and matching comparison cohort flow chart.

Our study identified and retrieved 10,857 patients newly diagnosed with RA from a total of 1,000,000 individuals sampled from the Taiwan NHIRD during the period from 2001 to 2009, as shown in Figure 1. The identification of patients with RA was based on the ICD-9-CM diagnosis code 714.0 and the presence of at least two outpatient services or one hospitalization for RA during the study period and after the initial date of diagnosis. The accuracy of RA coding in NHIRD had been validated in previous studies (24, 27). Patients were excluded whose first diagnosis of RA was during the year 2000, to guarantee a minimum follow-up time of at least 1 year. Patients were also excluded if they were diagnosed with any cerebrovascular diseases or received treatment for RA prior to the time of the first RA diagnosis. Not only does the Taiwan NHIRD record patients' disease diagnoses, it also tracks patients' prescription dispensing history. With our interests and the validity of results in mind, we selected those patients with continuous prescription history. Similar patient identification processes and methods were verified in previous studies (24, 27). Overall, 7,904 RA patients who were at least 18 years of age and followed up for at least 90 days were eligible for inclusion in this study. During the follow-up appointments, it was found that 660 RA patients had an ischemic stroke.

### Outcomes and covariates

The study endpoint was ischemic stroke as defined by the ICD-9-CM codes 433–437 (433: occlusion and stenosis of precerebral arteries; 434: occlusion of cerebral arteries; 435: transient cerebral ischemia; 436: acute, but ill-defined, cerebrovascular disease; and 437: other and ill-defined cerebrovascular disease, including cerebral atherosclerosis, non-ruptured aneurysm, Moyamoya disease). The baseline covariates Charlson comorbidity index (CCI), hypertension and hyperlipidemia were based on the outpatient information prior to the index date of RA diagnosis for each patient. Covariates are independent variables that may induce our endpoint of interest and should be identified to eliminate potential bias, therefore needing propensity score match (24, 27–31). Table 1 shows the demographics and clinical characteristics of patients, including gender, age and the baseline covariates of CCI, hypertension and hyperlipidemia at the initial diagnosis of RA.

**Table 1:** Cox proportional Hazard model for RA patients, showing the effect of baseline covariates.

	**Non-Celecoxib**	**Celecoxib**	***p* value**	**Non-Etoricoxib**	**Etoricoxib**	***p*-value**
Age			<0.001^***^			0.0038^**^
≥18 and ≤ 30	459 (6.88)	43 (3.48)		493 (6.42)	9 (4.04)	
>30 and ≤ 50	2,589 (38.82)	352 (28.50)		2,877 (37.46)	64 (28.70)	
>50	3,621 (54.30)	840 (68.02)		4,311 (56.13)	150 (67.26)	
CCI			<0.001^***^			0.6545
0	62 (0.93)	29 (2.35)		89 (1.16)	2 (0.90)	
1	6,049 (90.70)	1,074 (86.96)		6,918 (90.07)	205 (91.93)	
≥2	558 (8.37)	132 (10.69)		674 (8.77)	16 (7.17)	
Hypertension			<0.001^***^			0.777
No	5,073 (76.07)	853 (69.07)		5,757 (74.95)	182 (0.45)	
Yes	1,596 (23.93)	382 (30.93)		1,924 (25.05)	41 (81.61)	
Hyperlipidemia			0.1543			0.1763
No	5,673 (85.07)	1,031 (83.48)		6,522 (84.91)	182 (81.61)	
Yes	996 (14.93)	204 (16.52)		1,159 (15.09)	41 (18.39)	

*** p < 0.001;

** p < 0.01;

* p < 0.05; p < 0.1.

None of the baseline covariates has significant time-varying effects on ischemic stroke. The proportional hazards assumption is valid. The Cox model is appropriate.

HR, hazard ratio; CI, confidence interval; RA, rheumatoid arthritis; CCI, Charlson comorbidity index.

### Statistical analysis

Cox proportional hazard models were used to compare the risks of ischemic stroke through HRs and the corresponding 95% CIs. The follow-up duration was from the first diagnosis of RA to onset of ischemic stroke, withdraw from the National Health Insurance system, or December 31, 2010, whichever occurred first. The adjusted HR was included for age, gender, Charlson comorbidity index, hypertension, and hyperlipidemia. The Chi-square test was used to test for non-proportionality of hazards in the Cox model. When the proportionality assumption was violated, a spline curve of the scaled Schoenfeld residuals was fitted to demonstrate the estimated preventive effect on ischemic stroke over time for drug usage (32). Kaplan Meier curves were constructed to reveal the ability of covariates and drug usage to predict the risk of ischemic stroke. Dosages for Celecoxib were defined in the ranges of ≤ 200 and >200 mg/day while those for Etoricoxib were 0 and >0 mg/day. Due to the reduced gastrointestinal bleeding benefit of Celecoxib in comparison to other NSAIDs, Celecoxib is generally preferred in prescriptions for patients with RA (33). Since the typical dosage for Celecoxib is 100–200 mg twice a day, the ranges of ≤ 200 and >200 mg/day are suitable to distinguish and identify patients with higher dosages and their respective effects (34). Etoricoxib, on the other hand, shows better efficacy and is therefore usually prescribed for patients who need further assistance in analgesia. Setting the cutoff point at 0 mg/day could capture these aspects of patients and better compare Etoricoxib's pharmacological effects (35, 36). The non-parametric log-rank test was used to evaluate the importance of each risk factor. The statistical analyses in this study were executed using R software version 3.2.0 [R Foundation for Statistical Computing (http://www.R-project.org/), Vienna, Austria].

## Results

### Baseline characteristics of RA patients with ischemic stroke

Based on the selection criteria used in this study, Table 1 shows the demographics for the Cox proportional Hazard model and the respective baseline covariate effects among the subjects. Among the 7,904 RA individuals, 6,669 were not prescribed Celecoxib (*p* < 0.001) and 7,681 were not prescribed Etoricoxib (*p* = 0.0038). When further divided into subgroups, 459 individuals aged 18–30 years were not on Celecoxib and 43 were, while 493 individuals were not on Etoricoxib and 9 were. For the 30–50 years age group, 2,589 subjects did not take Celecoxib and 352 did. Similarly, 2,877 subjects were not taking Etoricoxib while 64 were. Lastly, for patients >50 years of age, the numbers of individuals not prescribed Celecoxib or Etoricoxib were 3,621 and 4,311, respectively. Those taking Celecoxib and Etoricoxib were numbered 840 and 150, respectively. All of these *p* values were < 0.001.

In terms of the CCI, 91 individuals had no comorbidities, 7,123 had 1 comorbidity and 690 had ≥2. Among the patients with a CCI score of 0, only 29 were prescribed Celecoxib (*p* < 0.001). Among those with a CCI of 1, 1074 were taking Celecoxib (*p* < 0.001). Those with two or more comorbidities had 132 patients on Celecoxib (*p* < 0.001).

Among the 7,904 patients, 5,926 did not have hypertension but had 853 individuals took Celecoxib (*p* < 0.001). The remaining 1,978 patients with hypertension had 382 individuals with Celecoxib (*p* < 0.001). The 1,200 individuals with hyperlipidemia did not have significant differences in those who were prescribed either Celecoxib or Etoricoxib.

### Effect of NSAIDs on risk of ischemic stroke in patients with RA

Table 2 shows the effects of dosage and time for RA patients who took Celecoxib or Etoricoxib. The follow-up duration was 10 years upon identification of patients with RA. Among the 7,904 patients with RA, 6,669 did not take Celecoxib, of whom 564 (8.46%) had an ischemic stroke event. Of the 597 individuals who took ≤ 200 mg/day, 58 (9.72%) experienced stroke. The adjusted HR compared to those who did not take Celecoxib was 0.78 (95% CI 0.60–1.03, Chi square test *p* = 0.170). Of the 638 patients who took >200 mg/day of Celecoxib, 38 (5.96%) eventually experienced a stroke. Compared to those who did not take Celecoxib, this group had an adjusted HR of 0.67 (95% CI 0.48–0.93), with a Chi square test *p*-value of < 0.001. The respective Log-rank test (Mantel-Cox test) *p*-value was 0.03.

**Table 2:** Cox proportional hazard model for risk of ischemic stroke among patients with rheumatoid arthritis according to the effect of a single drug.

	**No. of patients (*N* = 7,904)**	**No. of ischemic stroke events (*N* = 660)**	**Log-rank test (Mantel–Cox test) *p*-value**	**Crude (Unadjusted) HR and 95% CI**	**Adjusted HR and 95% CI**
**Celecoxib**					
None	6,669	564		Ref	Ref
≤ 200 mg/day	597	58		1.07 (0.82, 1.40)	0.78 (0.60, 1.03)
>200 mg/day	638	38	0.03	0.65 (0.47, 0.91)^*^	0.67 (0.48, 0.93)^*^
(0, 3.5)	638	7		0.23 (0.11, 0.49)^***^	0.22 (0.10, 0.46)^***^
(3.5, 7)	519	19		0.88 (0.55, 1.41)	0.80 (0.50, 1.29)
(7, 10)	259	12		1.96 (1.05, 3.66)^*^	1.85 (0.98, 3.50)
**Etoricoxib**					
None	7,681	654		Ref	Ref
>0 mg/day	223	6	0.0047	0.33 (0.15, 0.74)^**^	0.35 (0.16, 0.80)^*^

*** p < 0.001;

** p < 0.01;

* p < 0.05; p < 0.1.

Adjusted for gender, age, Charlson comorbidity index, hypertension and hyperlipidemia.

HR, hazard ratio; CI, confidence interval.

Taking the time effect into consideration, a total of 638 patients with RA took Celecoxib from 0 to 3.5 years, with 7 (1.10%) ischemic strokes recorded in this group, significantly lowering their stroke risk (adjusted crude HR 0.22, 95% CI 0.10–0.46, *p* < 0.001). For the 519 individuals who took this medication between 3.5 and 7 years, 19 (3.66%) reported strokes, with an increased risk, but still lower than for those not taking Celecoxib (adjusted HR 0.80, 95% CI 0.50–1.29). For the 259 patients who took Celecoxib for 7–10 years, there were 12 (4.63%) stroke events (crude HR 1.96 95% CI 1.05–3.66; adjusted HR 1.85, 95% CI 0.98–3.50).

The analysis of the value over time of taking Etoricoxib showed that 7,681 did not take the medication and 654 (8.51%) experienced a stroke event. Of the 223 patients who took Etoricoxib, 6 (2.69%) had strokes (adjusted HR 0.35, 95% CI 0.16–0.80, *p* < 0.05).

### Kaplan Meier survival curves for risk of ischemic stroke associated with the use of Celecoxib or Etoricoxib

**Figure 2:**
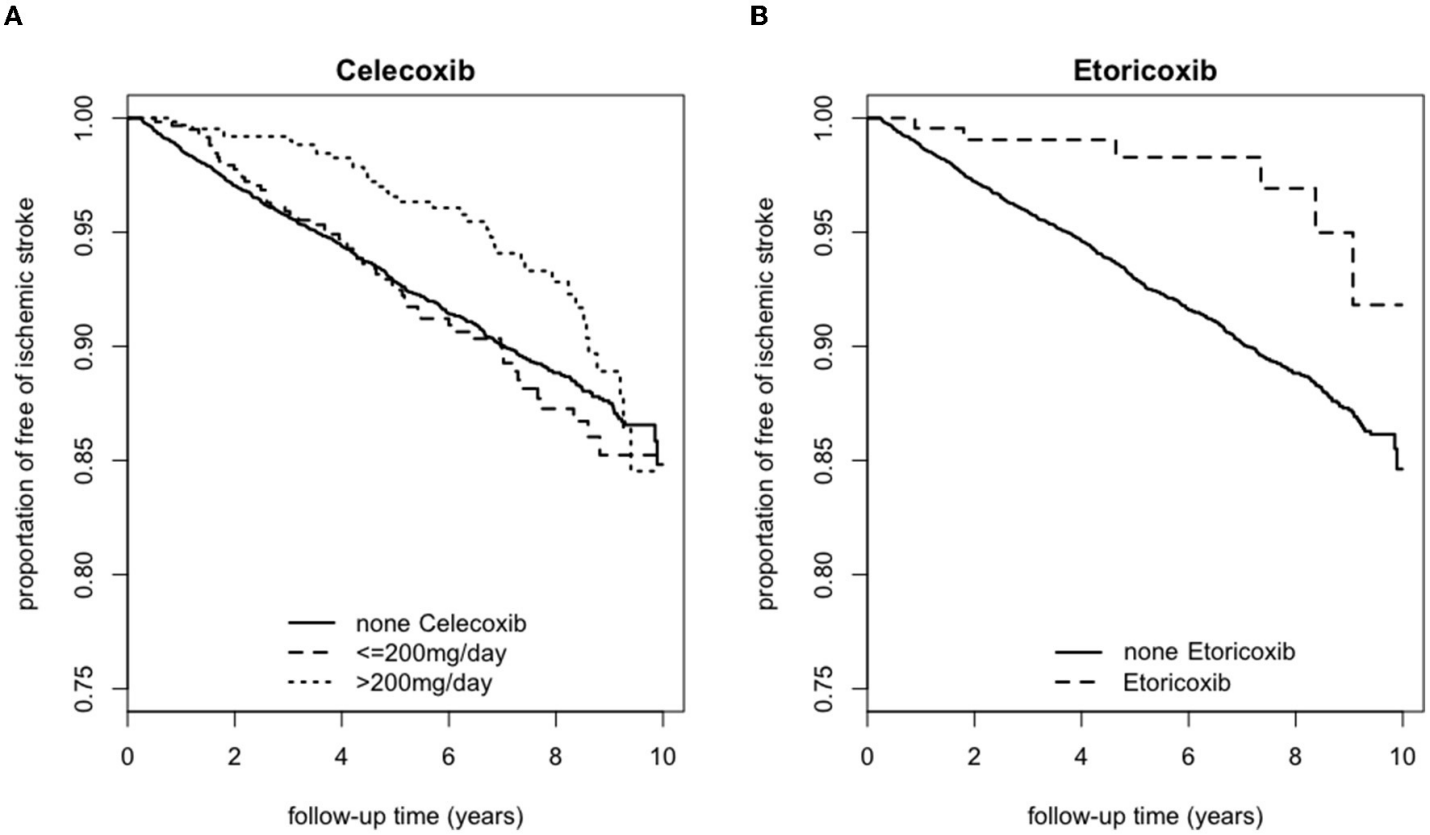
Kaplan Meier survival curve for those with ischemic stroke according to usage of **(A)** Celecoxib or **(B)** Etoricoxib over a 10-year follow-up period.

Figure 2 presented the Kaplan Meier survival rate for the 6,669, 597, and 638 individuals who were on (A) Celecoxib for 0, ≤ 200 and >200 mg/day, respectively, vs. the population that continued to remain free of strokes. In comparison with the baseline, those who took ≤ 200 mg/day of Celecoxib had a higher survival rate between 0 and 3 years. However, after the third year, the survival rate dropped slightly below that of those who did not take this medication. Individuals taking >200 mg/day generally were more likely to be free of strokes for the first 9 years. After that the survival rates among all three categories were comparable. The trends showed that patients taking (B) Etoricoxib were constantly more likely to be free of stroke during the 10 years of observation compared to those who did not take Etoricoxib.

### Time-varying effect of NSAIDs in ischemic stroke

**Figure 3:**
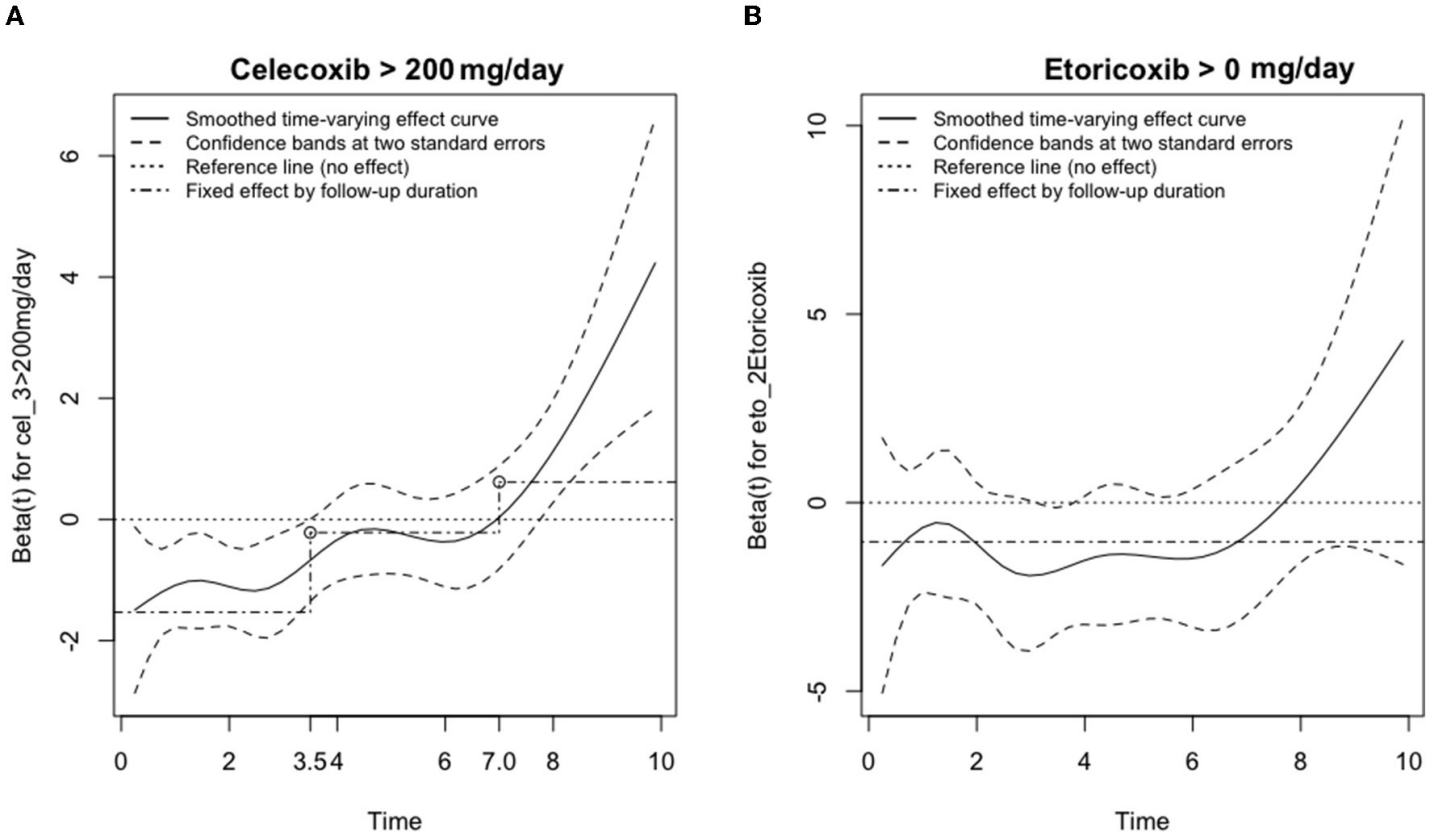
Adjusted effects on the risk of ischemic stroke for the usage of **(A)** Celecoxib or **(B)** Etoricoxib vs. time (in years). The smoothed time-varying adjusted effect curve is based on the scaled Schoenfeld residuals plot (solid), along with the confidence bands at two standard errors (dashed), the reference line (dotted) and the time-fixed effects estimated from the Cox model (dash-dotted) for NSAID dosage of **(A)** Celecoxib > 200 mg/day and **(B)** Etoricoxib > 0 mg/day.

Figure 3 presents the smoothed time-varying adjusted effect curved based on the scaled Schoenfeld residuals plot, along with the 95% confidence bands, the reference line and the time-fixed effects estimated from the Cox model for NSAID dosage. The first graph (A) shows that taking >200 mg/day of Celecoxib for <3.5 years provided a protective effect from strokes. When taken for between 3.5 and 8 years, the effect of Celecoxib on stroke probability was neutral. However, when Celecoxib was taken at this dosage for more than 8 years, the incidence rate for strokes drastically increased, indicating harm to the patients. The second graph (B) showed a similar effect for Etoricoxib. Before 8 years of taking Etoricoxib, the effects are neutral and may even be protective at 3.8 years. However, taking Etoricoxib for more than 8 years exponentially increased the probability of stroke.

## Discussion

Our study shows that a higher dosage of Celecoxib for a shorter amount of time (i.e., >200 mg/day for 0–3.5 years) lowers the eventual incidence of ischemic stroke. On the other hand, the dosage and duration effects of Etoricoxib are either neutral or slightly protective, as evidenced by the Kaplan Meier survival curve with a critical point at 8 years of consumption.

Individuals who are male, older, with hypertension, hyperlipidemia or higher CCI score have a significantly higher probability of experiencing an ischemic stroke. The rationale behind these baseline risk factors include the cellular inflammation associated with such diseases as RA (37). RA is, first of all, an inflammatory disorder. In a healthy individual and at baseline, the cellular construction of the synovium at a joint is comprised of a layer 1–3 cells thick; in an individual with RA, this lining can be 8–10 cells thick (38). The presence of hyperlipidemia and hypertension will elevate the level of lipid deposits in the bloodstream and increase the likelihood of occluded vessels. In hypertension, vasoconstriction increases the amount of pressure in the blood vessels. This pressure leads to endothelial cellular proliferation of the vascular muscle cells (39). With either hyperlipidemia or hypertension, the blood vessels become more occluded, thus making these conditions a primary risk factor for ischemic stroke.

NSAIDs reach therapeutic levels by affecting the equilibrium of agents such as prostaglandin and thromboxane, as well as the resulting cellular effects on cardiovascular function, platelet aggregation and smooth muscle proliferation (5, 37, 40). First, NSAIDs produce their pharmacological effects *via* inhibiting COX enzyme activity and preventing the conversion of arachidonic acid to prostaglandin G2. Since the overall expression of the prostaglandin biosynthetic pathway is determined by the activity levels of both COX-1 and COX-2, inhibiting enzyme expression yields a lower amount of prostaglandin products, therefore inhibiting smooth muscle proliferation, inhibiting platelet aggregation and ultimately decreasing inflammation and pain (41–45). On the other hand, thromboxane is a potent vasoconstrictor. Inhibition of thromboxane decreases the likelihood of occluded blood vessels, lowering the possibility of ischemic stroke (40, 46–50). Previous published studies proposed different hypotheses that due to the selectivity of COX-2 inhibitors, they do not affect thromboxane production due to its primary synthesis in platelets which only express COX-1 and therefore continue vasoconstriction (8, 12). However, they overlooked the effects of prostaglandin, which is cellular inflammation and pain. By reducing part of the mechanism that induces cardiovascular events, our hypothesis is that this would result in reduction of ischemic stroke, as evidenced by the data presented in our cohort study (40, 44, 46–50). Conversely, due to the reduction in prostaglandin production by COX enzyme inhibition, there might be related gastrointestinal side effects such as gastric ulcers. However, highly selective COX-2 inhibitors such as Celecoxib and Etoricoxib were researched and had shown to halve gastrointestinal toxicity side effects (23). Another study showed that dual inhibition of both COX-1 and COX-2 is the primary pathogenesis in gastric pathology and as a result COX-2 inhibitors alone do not appear to cause new gastrointestinal damage in humans (51). Subsequently, both medications of interest Celecoxib and Etoricoxib not only provide potential reduction in risk of ischemic stroke, but they also cause fewer gastrointestinal adverse events.

The data from Table 2 suggest that taking more than 200 mg of Celecoxib per day might be protective against ischemic strokes for patients with RA. Interestingly, for the patients who took Celecoxib for <3.5 years, this medication may have a protective effect, but the effect may be detrimental if taken for a longer period of time (HR 1.96, 95% CI 1.05–3.66, *p* = 0.120). The primary reason for utilizing HR in our study is identify whether or not either Celecoxib or Etoricoxib would predispose individuals to ischemic stroke events, as well as the respective risks for each dosage group. Since HR is the main and sometimes the only effect measure in epidemiological studies, it allows us to identify potential hazardous results based on exposure (52). This method had also been applied in previous retrospective studies (24, 27). In combination, taking more than 200 mg of Celecoxib for 0–3.5 years had the most protective effect against strokes. According to Gong et al., Celecoxib is quickly absorbed to reach peak concentration in the bloodstream at the 3-h mark and is extensively metabolized by the liver. The process of methyl hydroxylation forms hydroxycelecoxib as a result and is subsequently excreted *via* urine and stool (53). This leaves <3% of the original dosage. Therefore, an increased dosage of Celecoxib would have a more positive outcome. For Etoricoxib, taking any dosage significantly decreased the incidence of stroke. In terms of duration, while consuming Etoricoxib for 8 years might have neutral and potentially protective effects, similar to those shown at 3.8 years, a larger percentage of those who took Etoricoxib were shown to be free of strokes compared to those who never took it. This result indicates a neutral or potentially protective effect of Etoricoxib but the dose-dependent effect of Etoricoxib has yet to be discovered (54).

Based on Figures 2, 3, individuals who consumed Celecoxib had an initial protective effect of 3.5 years, followed by a neutral effect until 8 years, with an abrupt signal for detrimental outcomes onwards. This phenomenon might be attributed to Celecoxib's lower short-term effect on blood pressure (20, 55). In general, COX-2 inhibitors may increase blood volume and total peripheral resistance. Underlying mechanisms include inhibition of prostacyclin and further activation of endothelin-1 in kidneys, causing a rise in blood pressure (56). However, experimental studies did not show an alteration in blood pressure for individuals taking Celecoxib (56). While Celecoxib's true mechanisms are still unclear, our research team speculates that Celecoxib initially has a lower effect on renal functions. With long-term consumption of Celecoxib, adverse effects of COX-2 inhibitors gradually take place and increase blood pressure, potentially provide prothrombotic environment and raise the relative risk for ischemic stroke (53). With regards to Etoricoxib, even though Etoricoxib was approved for medical use in 2002, our study defined Etoricoxib prescription and consumption for newly diagnosed RA patients and followed up for 10 years thereafter. Therefore, we could still conduct our statistical analysis with confidence. Datapoints in Figure 2 presented trends that patients who took Etoricoxib were constantly more likely to be free of stroke throughout the 10 years of follow-up. However, the Kaplan Meier survival rate is an observation model. In order to more specifically compare the two groups, smoothed time-varying adjusted effect curved based on the scaled Schoenfeld residuals plot, along with the 95% confidence bands, are necessary to further analyze the two groups for statistical significance, as shown in Figure 3, which only shows neutral to slightly protective effects for up to 8 years. In addition, from years 8 to 10 of the Kaplan Meier survival curve, we noticed ~2.5% drop in patients free of ischemic stroke. This may also indicate that the detrimental effects of Etoricoxib took place and exponentially increased the probability of stroke.

In addition, while the previously mentioned patient-level meta-analysis by White et al. concluded that there is no difference in incidence of cardiovascular events between Celecoxib and non-selective NSAIDs, several points in the clinical trials do not match our study criteria (21, 57). First of all, the majority of these trials were <26 weeks in duration, offering limited insights. Secondly, among the 39 studies, 21 of them focused on patients with osteoarthritis and 8 more on ankylosing spondylitis, low back pain, and Alzheimer's disease, which deviate from our focus on RA. Lastly, patients who began on aspirin treatment remained in the study yet were categorized as non-users, thus potentially skewing study results.

The strengths of this retrospective cohort study include that the Taiwanese nationwide research database randomly selects data samples to monitor data accuracy. While selected subjects in this study might be prescribed with other medications, which is the nature of comorbidity and comprehensive healthcare, RA and non-RA patients were matched 1:2 with propensity score by age, gender, CCI, hypertension, hyperlipidemia and index date. These individuals had already been screened with rigorous criteria and were excluded if they were diagnosed with any cerebrovascular diseases or received related treatment prior to the time of the first RA diagnosis. Other medications not related to the topic of interest were not likely to contribute to unwanted results and therefore would not skew the outcomes. The method for this retrospective cohort study allowed a larger sample size that also controlled for baseline comorbidities to ensure a more accurate result. To further increase the plausibility, credibility, and reliability of our study, we also tracked patients' prescription dispensing history, which would suggest medication compliance. Similar patient identification processes and methods were verified in previous studies (24, 27). In addition, evidence of the possible beneficial effects of NSAIDs may improve future medical care of both RA and non-RA patients, particularly those at risk of ischemic stroke (25).

There are some limitations in this study. Firstly, misclassification bias is possible because we selected RA patients based on the ICD-9-CM code with the presence of two outpatient visits or one hospitalization for RA. Fortunately, the Taiwan National Health Insurance authority uses a peer-review auditing system to confirm the accuracy of diagnosis. Secondly, even though our study has a specific endpoint, which is ischemic stroke as defined by the ICD-9-CM codes 433–437, ICD-9-CM 437 includes other and ill-defined cerebrovascular disease. This brings potential ambiguity. However, due to the nature of patient screening and follow up for more than a year's time, our team had already excluded patients with prior diagnosis of cerebrovascular diseases. Therefore, any newly associated cerebrovascular outcomes were considered as our study endpoint. Thirdly, the study groups of both Celecoxib and Etoricoxib are those prescribed with these medications. For those not prescribed, they could potentially have underlying NSAID medications. In addition, the database lacks relevant lifestyle data such as the habits of smoking and drinking. To reduce this confounding issue, we did a propensity score match for lifestyle-related comorbidities, including diabetes, chronic obstructive pulmonary disease and hyperlipidemia to ensure baseline comparability. Furthermore, patient compliance may always be a confounding factor. Medical personnel could only continuously provide patient education every time the prescriptions are refilled. However, our research is unique in that this cohort study utilized Taiwan NHIRD that tracks patients' prescription dispensing records which minimizes recall bias and allows for longer duration of follow-up period (24). The paradoxal results of Celecoxib being protective at a higher dose in a shorter duration yet being detrimental in a longer duration may also need further investigations to understand its underlying physiological mechanism. Similarly for Etoricoxib, its neutral to slight protective effects are also paradoxal with currently unknown mechanism. Further clinical trials might be necessary to clarify causality, rather than simply the association between NSAID use and risk of ischemic stroke.

## Conclusion

This population-based retrospective cohort study has shown that Celecoxib and Etoricoxib may be associated with risk reduction of first-occurrence ischemic stroke in patients with RA in a dose- and time-dependent manner.

## Data availability statement

The original contributions presented in the study are included in the article/Supplementary materials, further inquiries can be directed to the corresponding author.

## Ethics statement

The studies involving human participants were reviewed and approved by Institutional Review Board (IRB) Approval Information: Approved by the Chung Shan Medical University Institutional Review Board, Number CS19009. The patients/participants provided their written informed consent to participate in this study.

## Author contributions

All authors had access to the data, contributed their interpretations to the discussion and collaborated in the development of the manuscript. All authors critically reviewed and provided feedback on subsequent versions. All authors made the decision to submit the manuscript for publication and vouch for the accuracy and completeness of the data and the fidelity of this report to the study protocol.

## Conflict of interest

The authors declare that the research was conducted in the absence of any commercial or financial relationships that could be construed as a potential conflict of interest.

## Publisher's note

All claims expressed in this article are solely those of the authors and do not necessarily represent those of their affiliated organizations, or those of the publisher, the editors and the reviewers. Any product that may be evaluated in this article, or claim that may be made by its manufacturer, is not guaranteed or endorsed by the publisher.

## Abbreviations

CCI: Charlson comorbidity index
CI: confidence interval
COX-2: cyclooxygenase-2 (COX-2)
HR: hazard ratio
ICD-9-CM, International Classification of Diseases, Ninth Revision: Clinical Modification
NHIRD: National Health Insurance Research Database
NSAID: nonsteroidal anti-inflammatory drug
RA: rheumatoid arthritis.
