# Supplementary material for: Celecoxib and Etoricoxib may reduce risk of ischemic stroke in patients with rheumatoid arthritis: A nationwide retrospective cohort study
Source: Front Neurol. 2022 Oct 20;13:1018521. doi: 10.3389/fneur.2022.1018521 (PMC9630581; doi:10.3389/fneur.2022.1018521)
Supplement: Supplementary file 1 [file Table_1.DOCX]

**Supplementary Table 1:** Cox proportional hazard model for risk of ischemic stroke among patients with rheumatoid arthritis according to the effect of NSAID and corticosteroids

|  | No. of patients | No. of ischemic stroke events | Crude (Unadjusted) HR and 95% CI | Adjusted HR and 95% CI |
| --- | --- | --- | --- | --- |
| NSAID |  |  |  |  |
| No | 2264 | 178 | ref | ref |
| Yes | 5640 | 482 | 0.81(0.68-0.97)* | 0.65(0.54-0.78)*** |
| Corticosteroids |  |  |  |  |
| No | 6025 | 536 | ref | ref |
| Yes | 1879 | 124 | 0.67(0.55-0.81)*** | 0.62(0.51-0.76)*** |

***p<0.001; **p<0.01; *p<0.05; .p<0.1

Adjusted for gender, age, Charlson comorbidity index, hypertension and hyperlipidemia.

HR: hazard ratio; CI: confidence interval.
