# Supplementary material for: Celecoxib and Etoricoxib may reduce risk of ischemic stroke in patients with rheumatoid arthritis: A nationwide retrospective cohort study
Source: Front Neurol. 2022 Oct 20;13:1018521. doi: 10.3389/fneur.2022.1018521 (PMC9630581; doi:10.3389/fneur.2022.1018521)
Supplement: Supplementary file 2 [file Table_2.DOCX]

**Supplementary Table 2:** Cox proportional hazard model for risk of ischemic stroke among patients with rheumatoid arthritis according to the effect of celecoxib, etoricoxib, and DMARDs

|  | Crude (Unadjusted) HR and 95% CI | Adjusted HR and 95% CI |
| --- | --- | --- |
| Celecoxib |  |  |
| none | ref | ref |
| ≤200 mg/day | 1.07(0.82, 1.40) | 0.80(0.61-1.05) |
| >200 mg/day | 0.65(0.47, 0.91)* | 0.69(0.49-0.97)* |
| Etoricoxib |  |  |
| none | ref | ref |
| >0 mg/day | 0.33(0.15, 0.74)** | 0.36(0.16-0.81)* |
| DMARDs |  |  |
| No | ref | ref |
| Yes | 0.69(0.57-0.83)*** | 0.93(0.77-1.13) |

***p<0.001; **p<0.01; *p<0.05; .p<0.1

Adjusted for gender, age, Charlson comorbidity index, hypertension and hyperlipidemia.

HR: hazard ratio; CI: confidence interval.

DMARDs: disease-modifying anti-rheumatic drugs.
